# Supplementary material for: Impact of the Structural Modifications of Potato Protein in the Digestibility Process under Semi-Dynamic Simulated Human Gastrointestinal In Vitro System
Source: Nutrients. 2022 Jun 16;14(12):2505. doi: 10.3390/nu14122505 (PMC9230451; doi:10.3390/nu14122505)
Supplement: Supplementary file 1 [file nutrients-14-02505-s001.zip › nutrients-1757750-supplementary.pdf]

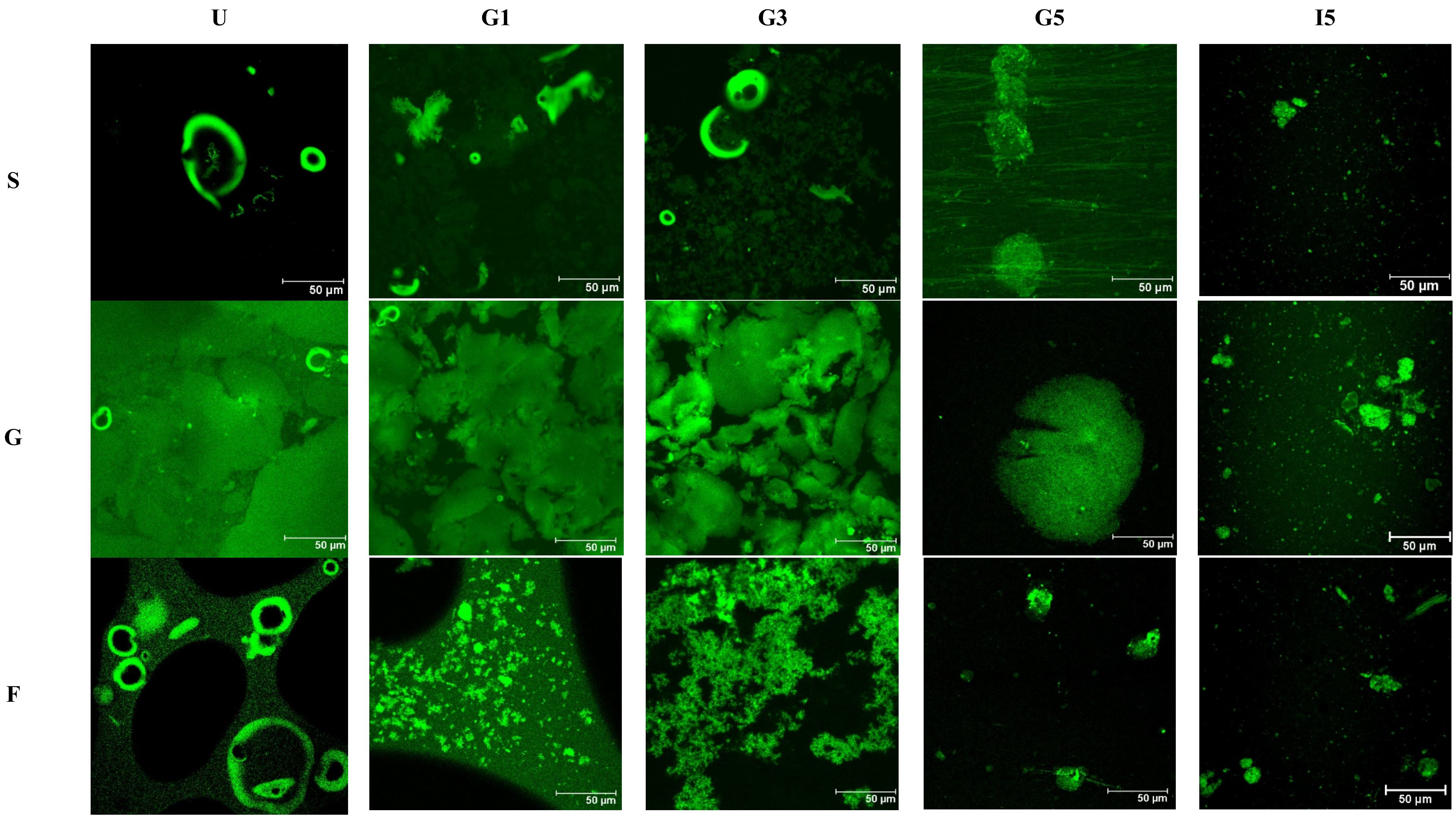

U
G1
G3
G5
I5
S
G
F
50 µm

HF

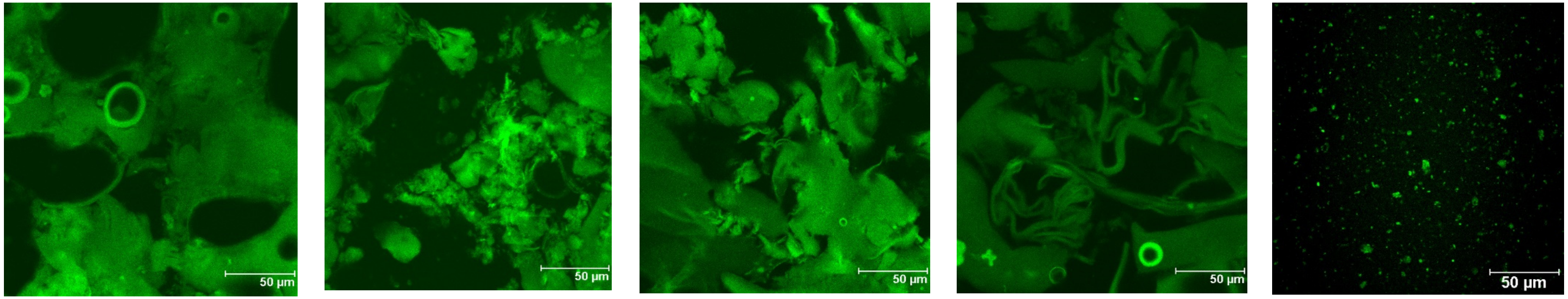

Figure S1. Confocal scanning laser microscopy (CSLM) of four different potato protein isolate structures (4% protein, w/w). Different gastric endpoints shown: GE1 (8.3 min), GE3 (24.8 min), and GE5 (41.3 min). Scale bar: 50 µm.

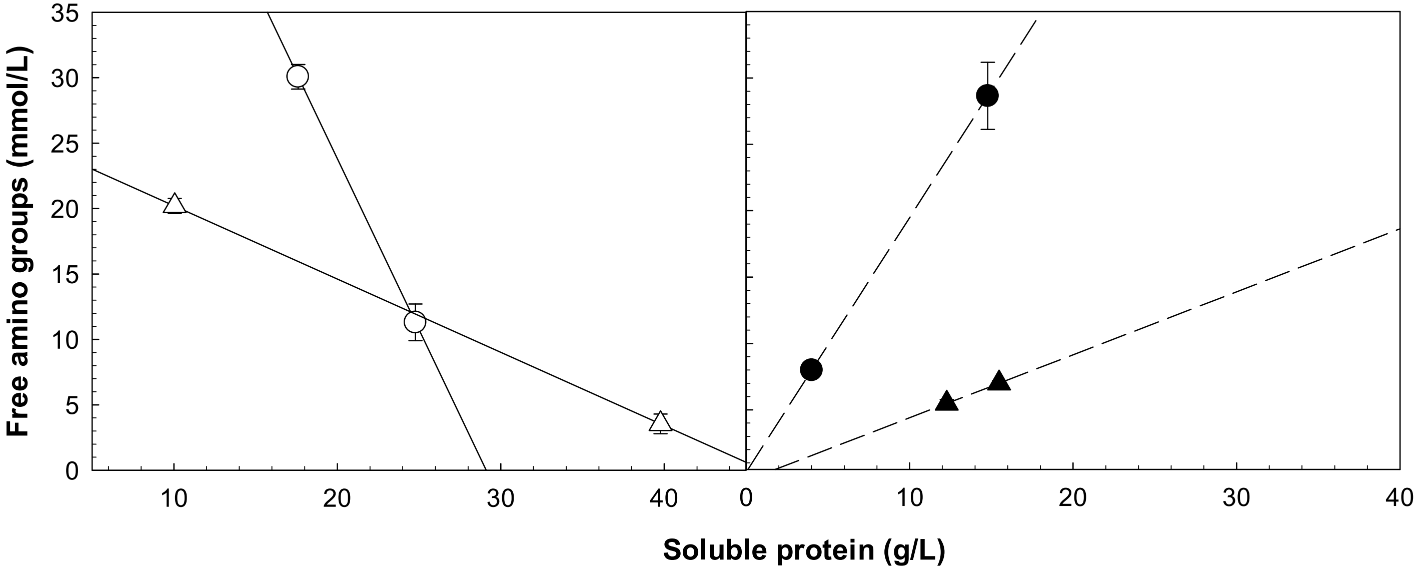

Figure S2: Amount of soluble protein and free amino groups (measured by OPA) for G1 and G5 fractions. A: unheated treatments, suspension (PoPI-S, empty circle), foam (PoPI-F, empty triangle); B: heated treatments, gel (PoPI-G, filled circle), heat-set foam (PoPI-HF, filled triangle). Lines are drawn to guide the eyes, arrows point from G1 to G5.

Figure S2 summarizes the difference between the first and last gastric emptying stage (G1 and G5) for unheated and heated treatments, showing the amount of soluble protein leaving the gastric stage and the amount of free amino groups. In all cases, the amount of free amino groups present in the soluble fraction of the gastric fractions increased between G1 and G5, but there were profound differences in the concentration of protein present in the fractions, due to differences in their emptying kinetics. In unheated samples (Figure S2A) the first gastric point showed higher values of soluble protein compared to G5, both for PoPI-S and PoPI-F. However, in PoPI-F (triangles) showed the highest amount of protein in G1. The heated samples showed a very different behavior (Figure S2B). The amount of soluble protein was much lower in G1 than in G5 and with lower values than those of unheated samples. In this case, PoPI-HF showed similar values of soluble proteins in G1 and G5, with low free amino groups, indicating a very low extent of digestion in the gastric stage. The level of soluble protein in PoPI-G was much lower at G1 compared to G5, once again demonstrating the important role of pepsin played in gastric digestion of potato protein isolate. More work is needed to clearly point to the differences in gastric emptying based on structural differences of the matrix, but the results in Figure S2 clearly indicate that structural changes caused the transit of different structures to the intestine. This had clear consequences to the extent of digestion in the intestinal phase (Figure 4), although the DH reached similar values (Figure 6).

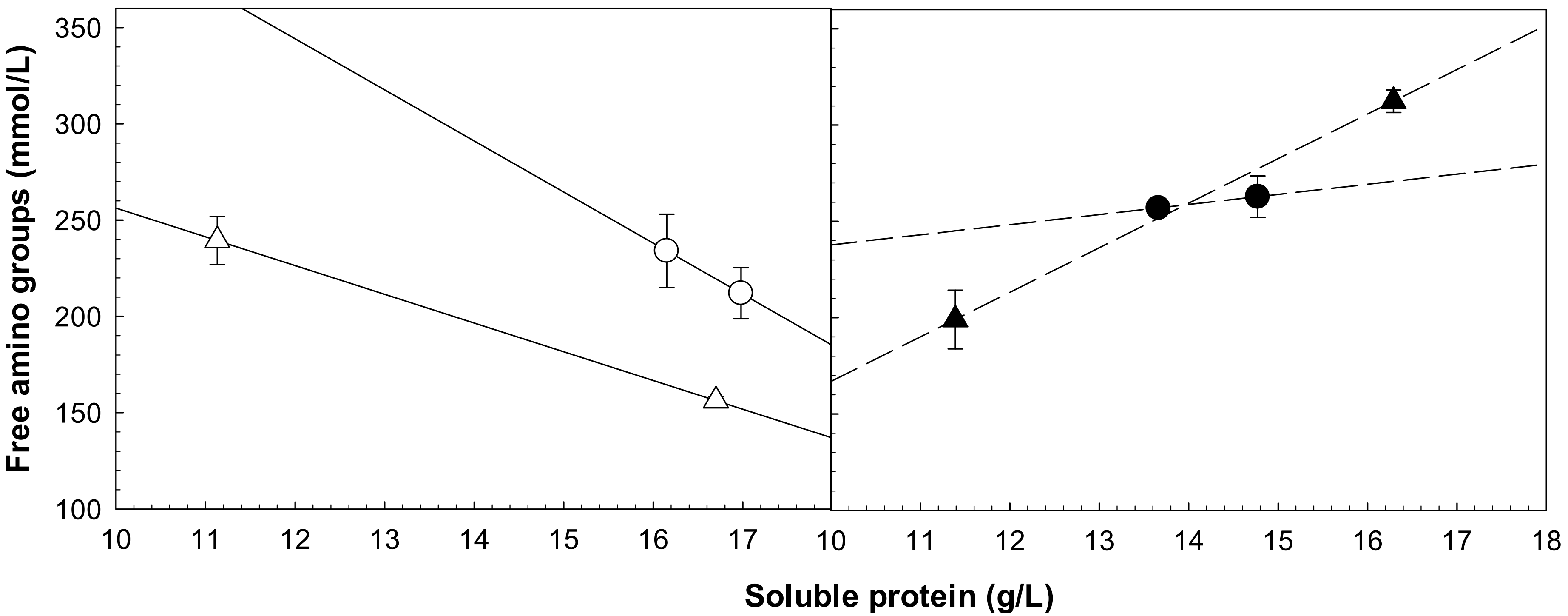

Figure S3. Amount of soluble protein and free amino groups for I1 and I5 fractions, corresponding to the . A: unheated treatments, suspension (PoPI-S, empty circle), foam (PoPI-F, empty triangle); B: heated treatments, gel (PoPI-G, filled circle), heat-set foam (PoPI-HF, filled triangle). Lines are drawn to guide the eyes, arrows point from G1 to G5.

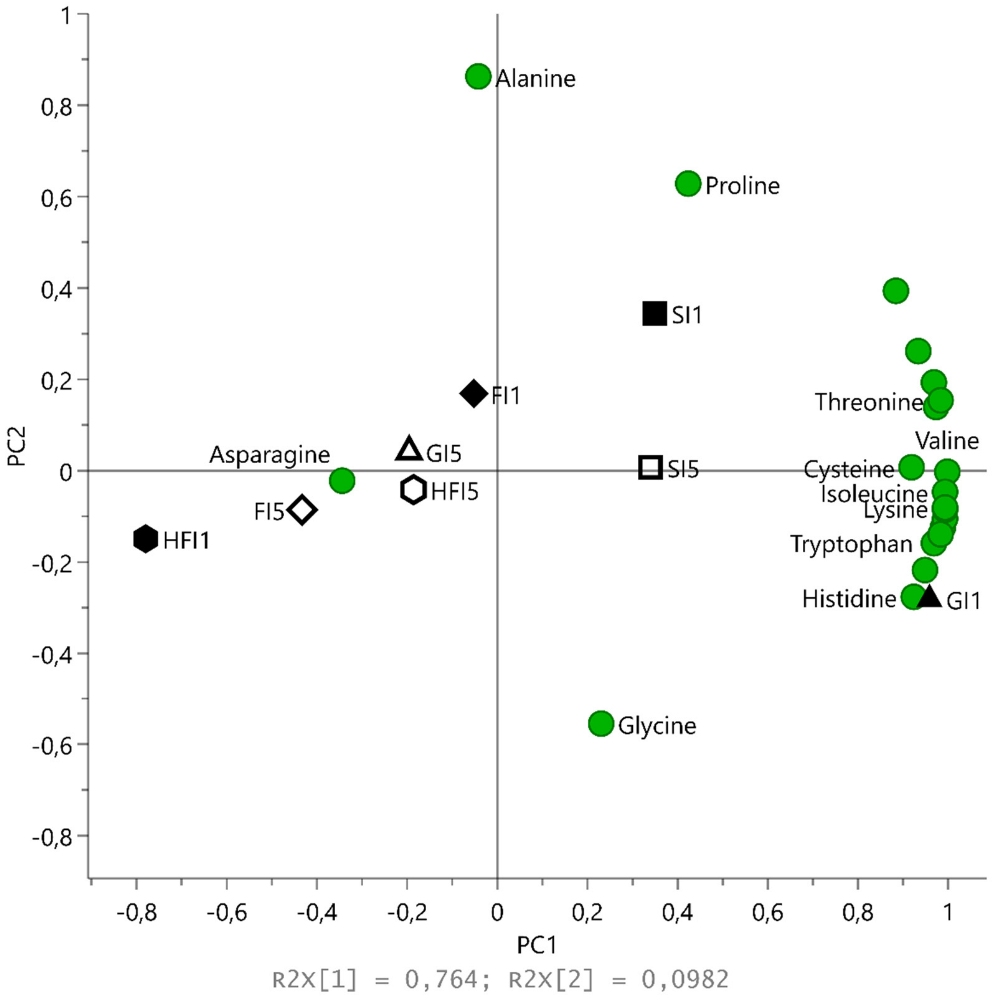

Figure S4. Principal component analysis (PCA) carried out with all free amino acids (see Table 1). Green circles indicate free amino acid values. Values chosen for the analysis correspond to *in vitro* intestinal digestion points: I1 (filled in black) and I5 (empty symbol). Potato protein isolate (PoPI) structures are represented with different symbols: Suspension (S, square), foam (F, diamond), gel (G, triangle), heat-set foam (HF, hexagon).

A principal component analysis including the release of FAA explains 86% of the data variation, in which 76% is explained by the first component and 11% is explained by the second. The average of two replicas for the four different potato protein structures at different intestinal points after 120 min of digestion (I1 and I5) are plotted in Figure S4. In the case of PoPI-S, both I1 and I5 are found closely clustered between each other. Similar values found between the intestinal points in the suspension indicate a constant release of FAA, most likely influence by a similar gastric emptying kinetics in these two points in which equal amounts of proteins are emptied and digested in a similar manner by proteases in pancreatin. Furthermore, the PoPI-S cluster is also close to the FAA cluster (to the far right) and second only after PoPI-G-I1. As previously mentioned, due to faster emptying of proteins.

Oppositely to PoPI-S, PoPI-G showed a high level of free amino acids in I1 due to the fast trypsin digestion and not to the fast gastric emptying, as previously shown in the degree of hydrolysis section. The data points of more complex structures (PoPI-F/HF are closely clustered to the far left, and showed a lower release of free amino acids, mostly due to slower gastric emptying. The PCA plotting allowed the understanding of the effect of structure complexity, gastric emptying and DH in the release of FAA at different intestinal points.
